# Jain point laparoscopic entry in contraindications of Palmers point

**DOI:** 10.3389/fsurg.2022.928081

**Published:** 2022-11-11

**Authors:** Nutan Jain, Sakshi Srivastava, Sri Lakshmi Prasanna Bayya, Vandana Jain

**Affiliations:** ^1^ Department of Obstetrics and Gynaecology, Vardhman Trauma and Laparoscopy Centre Pvt. Ltd., Muzaffarnagar, (UP), India; ^2^ Department of Gynae Endoscopy,Vardhman Trauma and Laparoscopy Centre Pvt. Ltd. Muzaffarnagar, (UP), India

**Keywords:** Jain point, laparoscopic entry port, left lateral port, non-umbilical entry, Palmer's point, laparoscopy in previous surgery, Hasson Technique, laparoscopic entry

## Abstract

**Background:**

This study was conducted to assess the efficacy of the Jain point to overcome the contraindications of Palmer's point. The Jain point lies on the left side of the abdomen at the L4 level, 10–13 cm lateral to the umbilicus. Due to its anatomical location, the Jain point is free from adhesions because postsurgical adhesions are encountered usually in the midline or the right side.

**Methods:**

This is a retrospective study conducted at a high-volume tertiary care referral center for advanced gynecological laparoscopic surgery, enrolling 8,586 patients who underwent laparoscopy at the center from January 2011 to March 2022. In this paper, we analyze 2,519 patients with a history of previous surgeries, who were operated using the Jain point.

**Results:**

In the 2,519 patients with a history of previous surgeries, the Jain point port was found to be adhesion free, regardless of the location of the scars, the number and type of previous surgeries, and those in whom Palmer's point was contraindicated. No major complications were reported, except for one case (0.04%) of small bowel injury, which was managed intraoperatively. The Jain point continued to function as the main ergonomic working port.

**Conclusion:**

The Jain point offers an alternate safe entry port in previous surgery cases for laparoscopic surgeons of various specialties, like general surgeons, urologists, oncologists, and bariatric surgeons, to overcome the contraindications of Palmer's point. The Jain point also acts as the main ergonomic working port, whereas Palmer's point becomes redundant after initial entry.

## Introduction

Adhesions require a highly evolved surgical intervention. Adhesions and their associated complications have piqued both the medical and the legal fraternity in recent years (1). Laparoscopic techniques have revolutionized the field of gynecological surgeries and have taken a significant leap ranging from diagnostic procedures to complex intricate surgeries. However, previous surgeries lead to adhesions, which significantly challenge safe entry in laparoscopy. Generally, first blind entry is through the umbilicus, which is the most common site for surgical adhesions. The incidence of intra-abdominal adhesions after laparotomy is 30%–90% (2). Most complications in laparoscopy occur during primary access when the trocar passes through the abdominal wall (3), and the rate is 0.4 per 1,000 cases for gastrointestinal injury and 0.2 per 1,000 cases for major vascular injury (4). This makes laparoscopic entry a significant burden on the healthcare system in previous surgery cases.

In 1974, R. Palmer devised a port that has acted as a savior in the aforementioned surgical situations (5); this lies 3 cm below the left subcostal margin in the midclavicular line and has been approved by experts across all subspecialties of endoscopy. Although generally safe, it has contraindications in cases of bloated stomach, upper abdominal masses, hepatosplenomegaly, and upper abdominal scars, causing a dilemma for laparoscopic surgeons, thus necessitating another entry port. The relatively newly devised ports such as the Lee–Huang point and 9th intercostal space are both in the upper abdomen, where the contraindications of Palmer's point persist.

**Figure 1:**
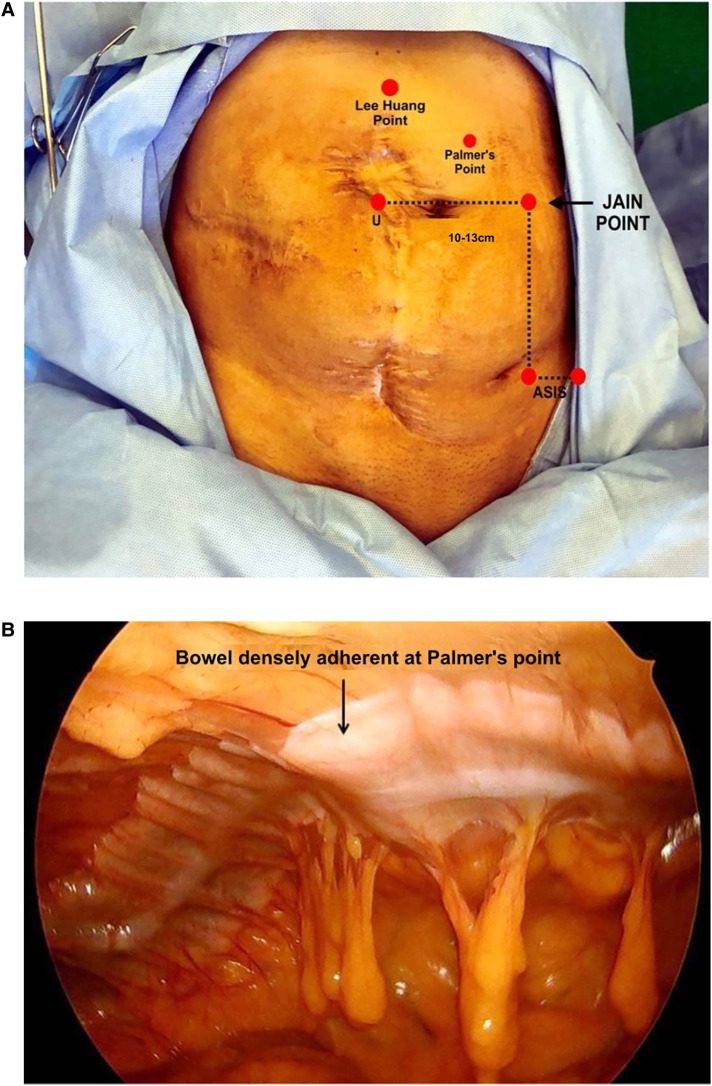
(**A**) Surface marking of a Jain point showing the distance from the umbilicus and the left lower port. The patient developed septicemia after surgery, following which multiple surgeries were done for colostomy and colostomy closure. (**B**) Finger pressing at Palmer’s point from the abdominal wall, showing bowel loops stuck over the upper abdomen. The Jain point avoids Type IIA bowel adhesions.

**Figure 2:**
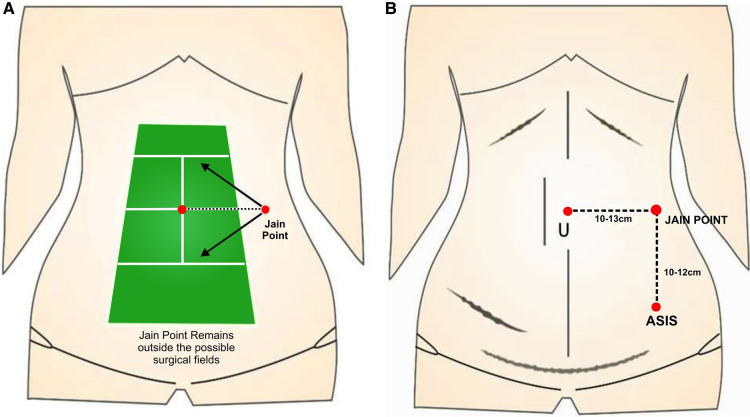
(**A**) Concept of a Jain point; it mimics the position of a referee on a tennis court who is sitting outside the surgical field. (**B**) Jain point is equally applicable for the upper-, mid-, and lower-abdomen scars.

The Jain point was devised in the mid-abdomen to avoid adhesions of the upper abdomen that contraindicate the previously described entry ports. The Jain point is 10–13 cm lateral to the umbilicus and outside the main surgical field avoiding the vessel, viscera, adhesions, and small and large bowel (Figures 1A,B). The Jain point, in effect, simulates the position of a referee on a tennis court, sitting outside at mid court, watching the movement of the ball on either side of the court with equal agility. In our context, it is outside the surgical field, and thus, applicable for scars in the upper, mid, and lower abdomen (Figures 2A,B) (6).

**Figure 3:**
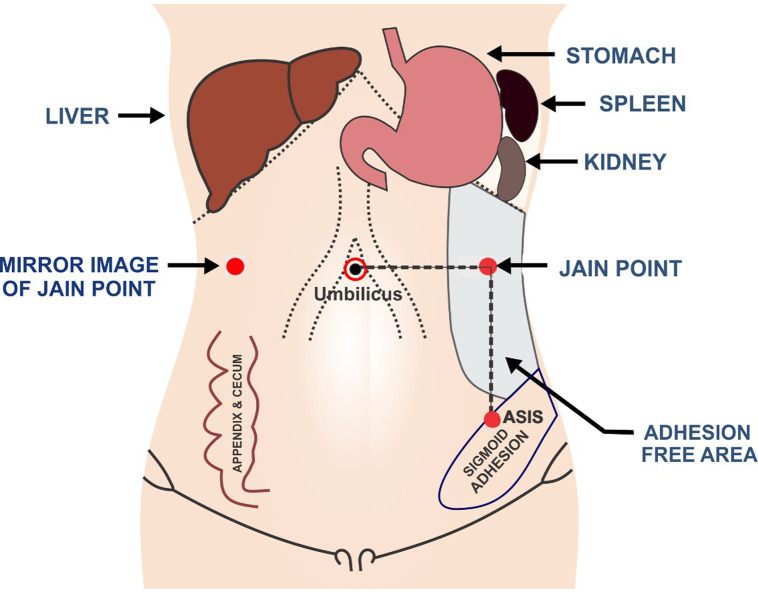
Jain point is at the L4 level, 10-13 cm lateral to umbilicus and away from both the viscera on the left side (spleen, kidney, and bloated stomach) and the sigmoid colon in a wide nascent area on the left side from L1 to pelvic, which is free from vessel, viscera, adhesions, and bowel.

**Figure 4:**
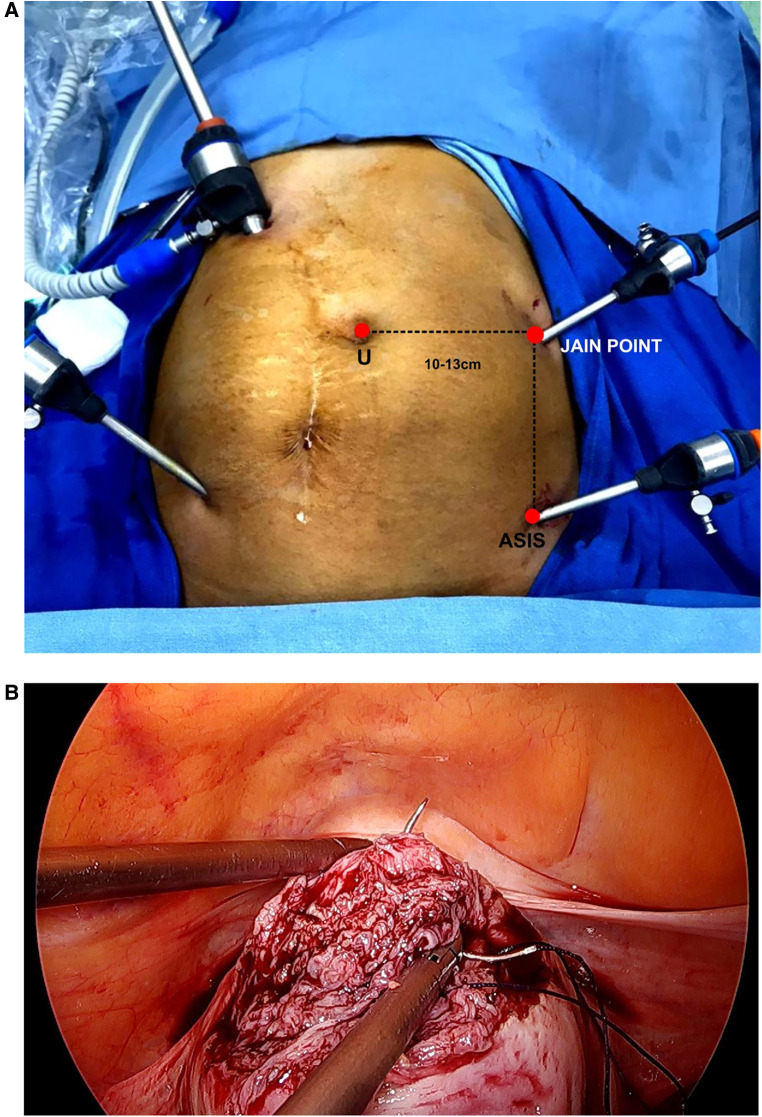
(**A**) 10 mm port inserted under the direct vision of the Jain point port, which becomes the main working port. (**B**) Ipsilateral ports making the myomectomy and suturing ergonomic.

Sharp (7), in his recent article in UpToDate titled “Overview of Gynaecological Laparoscopic surgery and Non-umbilical Entry Site”, strongly advocates non-umbilical entry in previous surgery cases, large pelvic masses, extremes of body mass index (BMI), pregnancy, very lax abdomen, and umbilical hernia. We adopted the concept of non-umbilical entry ahead of time as the Jain point is a non-umbilical entry port, at the L4 level,10-13cm lateral to umbilicus away from both, the viscera on the left side (the spleen, kidney, and bloated stomach), which are located higher up at T10 to L1, and the sigmoid colon, which has physiological adherence at the pelvic brim at the lower end. This leaves a wide nascent area on the left side from L1 to the pelvic brim, which is free from the vessel, viscera, adhesions, and bowel (VVAB) (8) (Figure 3) where the Jain point is located. The Jain point has the potential to avoid injury to the major retroperitoneal vessel (MRV). It also avoids superior epigastric and superficial epigastric vessels that are located within 5 cm of the midline. Located at the mid-abdomen, it reaches the depth of the pelvis or upper abdomen, and hence, continues to function as the main ergonomic working port throughout the surgery (Figures 4A,B).

This paper outlines the study of the Jain point as an alternate entry site in patients with a history of previous surgery and those in whom Palmer's point is contraindicated. For quick reference, we have tabulated the differences between the Jain point and Palmer's point (Table 1).

**Table 1:** Differences between Jain point and Palmer's point.

Primary port	Palmer's point	Jain point
Point proposed	1974	2011
Bony Landmarks	Two landmarks	One prominent bony landmark, i.e. the ASIS (anterior superior iliac spine)
	1. Mid clavicle	
	2. Subcostal margin	
Learning curve	Short	Short: eases the tension from beginner's mind
Time spent in port creation	Short	Short
Use as the main working port	No, becomes redundant after entry	Yes, becomes main ergonomic working port
Contraindications	1. Bloated stomach	No known contraindications
	2. Hepatosplenomegaly	
	3. Upper quadrant scars	
	4. Large upper quadrant masses	
	5. Suspected postinflammatory adhesions	
Entry from the right side	Cannot be made due to the risk of liver laceration	Can be used as a mirror image on the right side
Incidence of bowel injury in previous surgery	Injury to the bloated stomach likely but largely unreported	0.04%
Use in previous upper abdominal surgical scars	Not applicable in big upper abdominal scars	Can be used in upper abdominal scars
Use in cases with a history of infectious pathologies	Entry associated with the potential risk of visceral injury in case of upper abdominal adhesions	Safe entry in suspected upper abdominal adhesions
Use as first blind port routinely	Not reported	Reported by Jain et al.

The Jain point has a single very prominent bony landmark, the ASIS, which lies in the sterile surgical field, whereas Palmer's point is located by the clavicle which lies in the unsterile surgical field. Harry Reich, in his foreword for the book, “Non-Umbilical Laparoscopic Entry Ports”, published in the year 2020 (9), mentions that the Jain point with a prominent bony landmark is the lowest of all entry ports and may be best for routine use. The left lateral Jain point located in the mid-abdomen can be utilized as the main ergonomic working port for both the upper and the lower abdomen (10), whereas Palmer's point becomes redundant after initial entry. With the Jain point being away from the viscera, complications associated with postinflammatory patients suspected with a high adhesion score (classified as Type IIA adhesions) even without surgery do not occur (11). These cause omental and bowel adhesions affecting the upper abdomen, which can contraindicate Palmer's point. The Jain point can be used for all BMI patients, including low BMI patients (12) with previous surgery, whereas Palmer's point has limitations with low BMI patients due to its proximity to the left kidney (13).

## Material

We collected the data of 8,586 patients who underwent laparoscopic surgery at our center from January 2011 to March 2022. Their history and operative notes were carefully recorded. The patients’ age, BMI, indication of previous surgery (gynecologic or surgical), number of previous surgeries, mode of previous surgery (open or laparoscopic), and incisions of previous surgeries were tabulated. Complications were defined as events that significantly altered the planned procedure, deviated from the normal postoperative course, delayed discharge, or led to a prolongation of recuperation. Complications directly related to the entry technique that occurred intraoperatively, and discovered up to 2 weeks postoperatively, were recorded. Lastly, patients were followed up for any incisional hernia at the Jain point.

*Note*: To avoid inflation of data, cases of patients with multiple surgeries were counted only once when tabulating data for the mode of surgery, indication of surgery, and type of incision. For instance, if a patient had undergone laparotomy with a right paramedian incision and a cesarean section with a Pfannenstiel incision, only the right paramedian incision was considered, because the probability of having paraumbilical adhesions is more with this type of surgery and incision.

## Method

The preoperative preparation comprises a low residual diet for 48 h prior to surgery and mechanical bowel preparation in all previous open surgery cases. In patients with acute clinical conditions necessitating laparoscopic, diagnostic, or therapeutic procedures, Jain point entry is made without a long bowel preparation protocol after the anesthetist administers nasogastric tube. As many procedures in the study group have been done notably in ectopic pregnancies, T.O. abscess, and the torsion of the ovary, the surgery is performed under general anesthesia, with the patient lying in the dorsal lithotomy position. The entire abdomen is inspected and previous incision sites are noted. To locate the Jain point, the ASIS, which is a prominent fixed bony landmark in the sterile surgical field, is marked and a vertical line is drawn 2.5 cm medial to the ASIS up to the level of the umbilicus. Then, a horizontal line is drawn at the upper margin of the umbilicus. The point where the two lines meet is the “Jain point”. In effect, the entry point is located approximately 10–13 cm lateral to the umbilicus, depending on the patient's body type, BMI, and central obesity.

Notably, the abdominal wall is not lifted, avoiding unequal forces being applied during entry and zigzag track, providing a precise appreciation of layer-by-layer entry. The same technique is applied for thin or obese patients. The Veress needle is preferred, and it is entered perpendicular to the skin. Two pops are clearly heard, the first when the needle passes through the aponeurosis of the external oblique muscle and the second when the needle passes through a fused aponeurosis of the transverse abdominal muscle and internal oblique muscle, after which the needle encounters resistance as it enters the peritoneal cavity. Safety checks are done, the pneumoperitoneum is created, and the 5 mm trocar and telescope are inserted. The area below the entry point is inspected to rule out any injury to the bowel or vessel. Then, a 360° check of the abdominal cavity is done. A note is made of adhesions and their scoring and location, especially in the upper abdomen, to look for adhesions below Palmer's point and the right and left hypochondria to check for any subdiaphragmatic adhesions. Then, a 10 mm telescope is entered at an adhesion-free area according to the mandate of the surgery. The Jain point provides a working distance of 10–12 cm from the left lower port for good ergonomic working, becoming the main ipsilateral ports throughout the surgery. General surgeons and urologists have used them according to their convenience either as a camera port or as a working port later on in the course of surgery.

## Results

The results of the study spanning 11 years and 8,586 cases highlight the safety of the Jain point as a routine entry port in previous surgery cases. This paper is restricted to analyzing the results in 2,519 patients who had previous surgeries and the Jain point’s possible role in the contraindications of Palmer's point. The demographic profile of the patients, such as BMI, number of previous surgeries, types of scars, and indication of previous surgeries, is tabulated in Tables 2–5, respectively. The indication of previous surgery was found to be a very important factor to predict the adhesions preoperatively.

**Table 2:** Distribution of previous surgery cases according to body mass index.

BMI	Numbers
<18.5	83 (3.29%)
≥18.5 to <25	1,039 (41.24%)
≥25 to <30	960 (38.11%)
≥30 to <40	395 (15.68%)
≥40	42 (1.66%)
Total	2519

BMI, body mass index.

**Table 3:** Distribution of cases according to the number of surgeries in the past.

Number of previous surgeries	Prev. laparotomy	Prev. laparoscopy
Prev. 1 surgery = 1,907 (75.70%)	817	1090
Prev. 2 surgery = 457 (18.14%)	389	68
Prev. 3 surgery = 122 (4.84%)	116	6
Prev. 4 and more surgery = 33 (1.31%)	32	1
Prev. total cases = 2519	1,354 (53.75%)	1,165 (46.24%)

**Table 4:** Distribution of patients according to the type of scar of previous surgery.

Transverse scar	Vertical scar	Mc Burney	Kocher's scar	Lap scars	Other scars
828 (32.87%)	427 (16.95%)	50 (1.98%)	44 (1.75%)	1,165 (46.25%)	5 (0.2%)

Note: other scars: Chevron scar = 3, gunshot wound = 2.

**Table 5A:** Patient's profile depending on the type and indication of previous gynae surgery.

Previous Gynae surgery (*n* = 2121) (84.20%)		
Indications of surgery	Laparotomy (1168) (55%)	Laparoscopy (953) (45%)
LSCS	838	–
Ectopic pregnancy	92	79
Endometriosis	12	217
Myomectomy	72	158
Hysterectomy	19	18
Infectious pathologies	3	183
T.O. mass/abscess	22	52
Ovarian cyst	54	49
Mullerian anomalies	–	8
Infertility workup	8	149
Ligation	24	25
Others, including pelvic floor repair and Tubo tubal reanastomosis	24	15

LSCS, lower-segment cesarean section.

**Table 5B:** Patient's profile depending on the type and indication of previous general surgery.

Previous abdominal general surgery (*n* = 398) (15.79%)		
Indications of surgery	Laparotomy (186) (46.7%)	Laparoscopy (212) (53.26%)
Appendectomy	51	59
Cholecystectomy	43	84
Renal surgery	3	1
Intestinal obstruction	25	–
Intestinal perforation	7	–
Mesh hernia repair	9	4
Septicemia	6	–
Hirschsprung's disease	7	–
Colon pull-through		
High imperforate anus	5	–
Other	30	64

Table 2 shows the distribution pattern of the 2,519 patients with previous surgery, according to their BMI, which ranged from <18.5 to ≥40 kg/m^2^. Our study reveals that overall, in patients with a history of previous surgery, we operated on more patients in the obesity group (54%), superseding the normal BMI group (42%). Keeping with the global trend of obesity, a majority of the patients in our study had a high BMI, who included 960 overweight patients, 395 obese patients, and 42 morbidly obese, while only 83 (3.29%) were underweight.

Table 3 shows the distribution pattern of the 2,519 patients based on the number of surgeries in the past. A total of 1,354 patients had previous laparotomy and 1,165 had previous laparoscopy as the mode of surgery. The maximum number of patients in our study (75.7%) had one previous surgery. Of these, 817 patients underwent open surgeries and 1,090 laparoscopic surgeries. A total of 18.1% patients (457) had two previous surgeries. Among these, 389 had laparotomy and 68 laparoscopies. The percentage of patients who underwent multiple surgeries was 6.1, including 122 patients who had three previous surgeries, while 33 patients had a history of four or more surgeries. Thus, in the previous laparotomy group, we had almost 600 patients with two or more previous surgeries, while in the laparoscopy group, most had one previous surgery.

Table 4 shows the distribution pattern of patients based on the type of scar of previous surgery. A total of 32.8% (828) of the patients had previous incision as transverse. Some patients had a long horizontal incision at the level of the umbilicus, given during the neonatal period. A total of 16.9% (427) patients had vertical scars, with a few patients having non-classical incisions such as previous drain sites, colostomy sites, incisions for renal surgeries, and long vertical incisions extending from the epigastrium up to the pubic symphysis in prior surgery for intestinal obstruction, burst abdomen, septicemia, and other complex pathologies. We also had patients with atypical abdominal scar marks, with prior ectopia vesicae and Hirschsprung's disease in the early neonatal period. A total of 1.98% (50) patients had a Mc Burney scar, 1.7% (44) had Kocher's scar, and 3 had Chevron incision, while 2 had bizarre incisions related to gunshot wound surgery. A total of 46.25% (1,165) patients in our study had previous laparoscopic entry ports at multiple sites.

Table 5: We split the previous surgery patients into those with gynae indication (Table 5A) constituting 84.2% (2,121 out of 2,519) and general surgery cases (Table 5B) constituting 15.79% cases (398 out of 2,519).

Table 5A: In the gynae group, open procedure with 55% (1,168 out of 2,121) was 10% more prevalent than the laparoscopic procedure with 45% (953 out of 2,121). In the laparotomic approach, previous cesarean sections constituting 71.74% (838 out of 1,168) were the most common, followed by ectopic pregnancy (8%, i.e., 92 out of 1,168), both of which had a substantial rate of adhesions. Endometriosis (1%) and myomectomy (6%) were a formidable group for readhesion formation and *de novo* adhesions. Dense adhesions at the entry point and the surgical site were noted in previous hysterectomy patients (1.6%) presenting for prolapse and posthysterectomy adnexal masses, ovarian remnant syndrome, and ovarian residual syndrome. Patients with previous surgery for infectious pathologies such as genital Koch's, septic abortion, and pelvic inflammatory disease had very advanced adhesions.

Table 5B: General surgery patients, in contrast to the gynae group, underwent more previous laparoscopies (53.26%, 212 out of 398) than previous laparotomies (46.7%, 186 out of 398) done by the open procedure. Cholecystectomy and appendectomy were the most common indications. We operated upon 13 patients with mesh hernia repairs, 7 with Hirschsprung’s disease, and 5 with a high imperforate anus, which were managed in infancy by colostomy and then by the colon pull-through procedure. We encountered scars going from the pubic symphysis to the xiphisternum in midline laparotomies for indications such as Koch's abdomen [the center is located in North India, which has the world's highest prevalence of Koch's (14–17)], which, at times, necessitated bowel resection and reanastomosis and second surgery for colon pullback. These patients had a high score of adhesions. In patients in whom there were multiple scars and/or drain sites on the left side, a mirror image of the Jain point was made from the right side where there is no risk of liver injury.

No significant entry-related complications were reported in the study with the use of the Jain point as the primary port. We encountered minor complications like preperitoneal insufflation or omental emphysema, although failed entry was a less-encountered problem. In some patients with omental adhesions involving the entire abdomen, a few entries were made through the omentum, which did not incite any bleeding or require additional interventions like suture or coagulation. No vascular injury was encountered intraoperatively, no surgery was converted to laparotomy, and no mortality was noted. There was no incidence of hematoma formation after surgery, and no incisional hernia was noted at the Jain point in the long-term follow-up.

Major complications were restricted to one case (across all 8,586 patients) of small bowel entry in a patient with transverse scar exactly at the level of the umbilicus due to a laparotomy performed in childhood. We strongly suspected bowel adhesions and performed an MRI, which, however, failed to report bowel loops underneath the scar. This complication was recognized immediately, the bowel loop was extracted by widening the port, and the bowel was sutured and reposited. The patient did well postoperatively and was discharged in 3 days. The major complication rate in our study of 2,517 cases of patients with previous surgeries is 0.04%, and the overall rate for the entire number of patients entered by using the Jain point is 0.011%.

About one-third of the entries were made by fellows and trainees, with 8–10 initial cases under the supervision of senior consultants, and the remaining completely independently, demonstrating the short learning curve and reproducibility of the procedure (Table 6).

**Table 6:** Entry made through Jain point.

Entry made by	No. of cases	Percentage
Senior consultant	2,978	34.68
Junior consultant	2,846	33.14
Fellows	2,762	32.16

## Discussion

Palmer advocated the insertion of the Veress needle three centimeters below the left subcostal margin in the midclavicular line (called Palmer's point) in previous surgery cases. Palmer's point has enjoyed many years of use worldwide by all practitioners of endoscopic surgery, like gynecologists, general surgeons, or urologists. But as the complexities of surgical indications and the number of previous surgeries have increased, many contraindications of Palmer's point have emerged, such as hepatosplenomegaly, portal hypertension, gastropancreatic masses, huge gynecological masses forming in the upper abdomen, and upper abdominal scars on the left side due to previous splenic or gastric surgery. Even in patients with no previous surgery, making an entry through Palmer’s point is challenging in those with Type IIA adhesions due to previous infectious pathology leading to bowel and omental adhesions in the upper quadrant of the abdomen, as encountered in Koch's abdomen (18–21), and previous septicemia.

To overcome these limitations, a few other non-umbilical ports were designed, namely the Lee–Huang point (22) and left 9th intercostal space (23). The Lee–Huang point was primarily devised for para-aortic lymph node dissection. Lying midway between the xiphoid process and the umbilicus, it had the benefits of higher location and central vision and a wider working space, thereby avoiding periumbilical adhesions. Although this point was extensively used in patients with previous surgeries, it was not free from complications. In a series of 188 cases, 2 omental injuries from primary port insertion and 1 colon injury were reported. Similarly, the entry point from the left 9th intercostal space in the anterior axillary line at the superior surface of lower rib was also used for Veress needle insertion. It had a failure rate of 0.39%, and the risk of splenic injury and bleeding from subcostal artery limited its use (24). Although these points had their advantages, being located in the upper abdomen, they shared the same contraindications as Palmer’s point (25).

The open Hasson technique is widely practised to remove the adhesions of previous surgeries. In this technique, blunt trocar is introduced through the vertical incision at the center of the umbilicus and pneumoperitoneum is created. Although this method took into account error-free controlled entry into the abdominal cavity, it failed to tackle the Type IIA adhesions where the bowel was densely adherent to the parietal peritoneum. A meta-analysis of 5,284 patients undergoing operative laparoscopy through this method reported primary access injuries, 1 bowel injury, 21 wound infections, 4 minor hematomas, and 1 umbilical hernia (26). It also has drawbacks such as a longer time to create a port and difficulty in maintaining the pneumoperitoneum during surgery due to leakage of gas. Also, its use is not well defined in patients with extreme BMI, becoming technically difficult to reach the rectus sheath with increased abdominal fat (27, 28).

The Jain point is shown to be safe for entry in patients with upper abdominal scars where entry through Palmer's point was not feasible. We reported one notable case of a patient with a large upper abdomen, in whom Chevron incision® was made where Palmer's point was contraindicated and entry was made using the Jain point (29). We reported another study containing 106 cases of patients with scars in the upper abdomen, where entry was made through the Jain point (30). In that study, the bowel was found stuck completely over Palmer's point in several patients with previous infectious pathologies (31), almost making a second layer of the peritoneum (Figure 1B). In these patients, the Jain point was used for laparoscopic entry and found to be free from adhesions. In a few patients, omental adhesions were noted, but these did not incite any bleeding during the entry of a 5 mm port and therefore did not necessitate a suture or cauterization.

In our study of 8,586 cases, the complication rates were 0.01% overall and 0.04% in previous surgery cases, across all age groups (8–76 years) and BMI ranges (12.66–58.11 kg/m^2^), which were lower than those in the aforementioned studies. This can be explained by the Jain point's lateral location on the left side, 10-13 cm from the midline, inherently avoiding MRV injury. The Jain point is located at the L4 level, while all the viscera, namely, the stomach, spleen, and kidney, come up to the T12 L1 level, leaving a large nascent area till the pelvic brim where the sigmoid colon adheres. Even an adherent and distended sigmoid colon remains much lower for it to become vulnerable to trocar injury from the Jain point. Surgeons making primary entry do not encounter small or large bowel adhesions and the abovementioned formidable structures. It is very important to stay far lateral, and only being paraumbilical does not guarantee complete safety against bowel adhesions.

Unlike Palmer's point, the Lee–Huang point, and the 9th intercostal space that become redundant after initial entry, the Jain point can be used as an ergonomic working port throughout the surgery as noted by HT Sharp. Beyond just gynecologists, the point is even more versatile for general, bariatric, and oncosurgeons who commonly operate on patients having scars in all quadrants of the abdomen. The Jain point is used as a camera or working port (32), as in cholecystectomy, appendicectomy, and evaluation of patients with pain in the abdomen and hernia repairs. It can be valuable in bariatric surgery as the study indicates the Jain point's safety in high BMI patients. Oncosurgeons, while using it as a primary procedure or with second-look procedures after neoadjuvant chemotherapy, have found it safe in the context setting of advanced adhesion scores in such indications (33). The mirror image of the Jain point can be used from the right side without incurring the risk of liver laceration, making it a viable port in the lateral position for urological procedures and, in general, surgical indications where scars or drain sites are present over the Jain point on the left side.

The Jain point has a short learning curve and is easily reproducible, due to the ease of locating it by a single prominent bony landmark, the ASIS, and an easy insertion technique, as demonstrated in our study, where one-third of laparoscopic entries were made by endoscopy fellows.

The Jain point has been referenced by researchers and laparoscopic surgeons in publications on laparoscopic entry noting the benefits of the Jain point in previous surgery cases. Wasson et al. (34) recommend the use of the Jain point in patients in whom adhesive disease is suspected. Bedaiwy et al. (35) have documented the benefit of the Jain point, particularly in patients with suspected periumbilical adhesions, to enhance the safety of the subsequent 10 mm supra-umbilical port entry. Einarsson et al. (36), in a recent textbook of Minimally Invasive Gynecology, describe the Jain point as an alternative to Palmer's point on the left side mid-abdomen, 2.5 cm medial to the ASIS. Reynolds (37) has highlighted in his thesis that the Jain point is proposed as an alternate entry site when the first three options (umbilicus, Palmer's point, and the Lee–Huang point) are not viable. Eamudomkarn et al. (38), in their recent publication, have cited the benefits of the Jain point over other points, as it is located lower and lateral than Palmer's and Lee–Huang points, avoiding injury to the viscera. Salcedo et al. (39) have described the use of the Jain point in laparoscopy in pregnancy, which is in line with our observation of over nine such surgeries done in pregnancy, where the feature of laterality gives more space for the gravid uterus.

Some publications such as “Clinical Perspective Concerning Abdominal Entry Techniques” by Recknagel et al. (40) and “Abdominal entry in laparoscopic surgery” by Pepin (41) mention the Jain point as a viable entry port in previous upper abdominal scars and previous surgery patients but have inaccurately depicted the Jain point as being located “directly lateral to the umbilicus, and 2.5 cm medial to a line drawn vertically upwards from left ASIS”. Contrarily, the Jain point derives its safety features by staying 10–13 cm lateral from the umbilicus where the risk of bowel adhesions is mitigated and it avoids injury to the superior and superficial epigastric vessels that lie within 5 cm of the midline. It is important to mark the surface of the Jain point properly by locating the ASIS and the remaining 2.5 cm from it and drawing a vertical line upward at the level of the umbilicus to gain the maximum benefits of safety and ergonomics. To further increase the safety of the Jain point, entry can be made through optical trocars that clearly show the different layers of the abdominal wall and parietal peritoneum as entry is made, and they can also detect bowel injury (42). To obtain a view before entry, disposable shielded trocars, called Ternamian™ threaded trocars (43), can be used. Mulayim et al. (44) have reported direct trocar entry from the Jain point. Reusable trocars have been reported to be safe in our series. How one makes an entry through this point is a matter of personal choice or institutional practice norms.

In conclusion, this study demonstrates the safety of the Jain point as a first blind entry port across all age groups and BMI ranges, with a low complication rate of 0.04% for previous surgery cases and an overall low complication rate of 0.01%, significantly lower than those in similar studies for other entry ports. The Jain point has no known contraindications and can be used by gynecologists, urologists, oncologists, and general and bariatric surgeons to overcome the contraindications of Palmer's point.

## Strength of the study

Sample size is a crucial consideration for quality research. This study includes 8,586 cases recorded over 11 years, and none of the previous studies have contained such a large number of patients and such a large time span.

## Limitation of the study

Although we have a large case series and a study period of 11 years, our study is retrospective in nature.

## Conclusion

Due to its anatomical location, the Jain point is a feasible entry option with low complication rates in situations where other ports have limitations. The port is safe for all types of previous scars in the upper, middle, and lower abdomen and can be used in all ranges of BMI. As per surgical need it can be used as a mirror image from the right side. Located in the mid-abdomen, it doubles up as the main ergonomic working port. It has no known contraindications. The Jain point can be used as an entry port for all practitioners of laparoscopy in previous surgery cases of patients in whom Palmer's point is contraindicated.

## Data Availability

The raw data supporting the conclusions of this article will be made available by the authors without undue reservation.
